# MicroRNAs: Clinical Relevance in Colorectal Cancer

**DOI:** 10.3390/ijms161226080

**Published:** 2015-11-25

**Authors:** Joe Thomas, Masahisa Ohtsuka, Martin Pichler, Hui Ling

**Affiliations:** 1 Department of Experimental Therapeutics, The University of Texas MD Anderson Cancer Center, Houston, TX 77054, USA; thomjoea@iupui.edu (J.T.); MOhtsuka@mdanderson.org (M.O.); martin.pichler@medunigraz.at (M.P.); 2 Division of Oncology, Medical University of Graz, 8010 Graz, Austria

**Keywords:** colorectal cancer, miRNA, pathophysiology, diagnosis, prognosis, treatment

## Abstract

Colorectal cancer is one of the most common cancer diagnoses and causes of mortality worldwide. MicroRNAs are a class of small, non-coding regulatory RNAs that have shown strong associations with colorectal cancer. Through the repression of target messenger RNAs, microRNAs modulate many cellular pathways, such as those involved in cell proliferation, apoptosis, and differentiation. The utilization of microRNAs has shown significant promise in the diagnosis and prognosis of colorectal cancer, owing to their unique expression profile associations with cancer types and malignancies. Moreover, microRNA therapeutics with mimics or antagonists show great promise in preclinical studies, which encourages further development of their clinical use for colorectal cancer patients. The unique ability of microRNAs to affect multiple downstream pathways represents a novel approach for cancer therapy. Although still early in its development, we believe that microRNAs can be used in the near future as biomarkers and therapeutic targets for colorectal cancer.

## 1. Introduction

Colorectal cancer (CRC) is currently the third most prevalent cancer type worldwide and is a leading cause of cancer mortality for both men and women [1,2]. It was estimated that in the United States in 2014 more than 130,000 cases of CRC were newly diagnosed and more than 50,000 deaths occurred due to this disease [2]. Despite the fact that the incidence rate of CRC has been steadily declining, owing at least partially to improved diagnostic and therapeutic options, and decreased societal risk factor exposure, the five-year survival rate for CRC is an unsatisfactory 64.9%, highlighting the need for innovative detection and treatment applications [2,3,4].

MicroRNAs (miRNAs) are a class of small, non-coding RNA molecules that have been shown to play an important role in the pathogenesis of several human diseases, including cancer [5]. These RNA molecules are around 20–22 nucleotides in length and act to modulate the expression of other genes [6]. Initially discovered as an important regulator in the development of *C. elegans* in 1993, a surge of subsequent research has shown the importance of miRNAs in almost every aspect of physiological and pathological conditions [7,8]. miRNAs exert their effects through complementarity binding to the 3′ untranslated region (UTR) of target mRNA sequences, resulting in the subsequent degradation of mRNA or repression of mRNA translation into protein [8]. The biogenesis of miRNAs involves a complex process including multiple stages. First, RNA polymerase II synthesizes primary miRNA (pri-miRNA) in the nucleus, which undergoes cleavage by DROSHA and its cofactor DGCR8 to yield the premature miRNA strand (pre-miRNA). Pre-miRNA is then transported out of the nucleus into cytoplasm via Exportin-5, where it undergoes further processing by DICER to yield the miRNA duplex. The duplex unwinds and the mature single-stand miRNA is incorporated into RISC (RNA-induced silencing complex), through which it exerts its regulatory effects on mRNAs [8]. The functional significance these molecules may have is further illustrated by the fact that a single miRNA can target multiple genes for repression, and multiple miRNAs may target the same gene [9]. Findings suggest that the total number of miRNAs in the human genome is 2588 [8]. Due to the widespread importance miRNAs play in human developmental processes, it is not surprising that they have important implications with regards to tumorigenesis. For example, it has been shown that miR-15 and miR-16 are downregulated in chronic lymphocytic leukemia (CLL), let-7 is downregulated in breast and lung cancers, miR-127 expression is silenced in bladder cancer, and miR-21 is overexpressed in glioblastomas [5,10,11]. These are but a few examples, and numerous other associations between miRNAs and human cancers have been found.

The strong correlation between miRNA expression and tumorigenesis has resulted in a great deal of research looking into their potential utilization as cancer biomarkers and therapeutics. Altered miRNA levels have also been associated with the maintenance of cancer stem cells (CSCs), angiogenesis, and epithelial-mesenchymal transition (EMT), all of which contribute to malignancy [12,13,14]. In this review article we describe the pathophysiology of miRNAs in CRC, point to their potential uses as diagnostic and prognostic biomarkers, and describe their potential role in innovative therapeutics. This paper represents a concise version for clinicians who will benefit from a short yet comprehensive clinically relevant review. This is also the first review to our knowledge that incorporates miRNAs showing importance in ulcerative colitis-associated CRC (UC-CRC).

## 2. MicroRNA (miRNA) Involvement in Colorectal Cancer (CRC)

**Figure 1:**
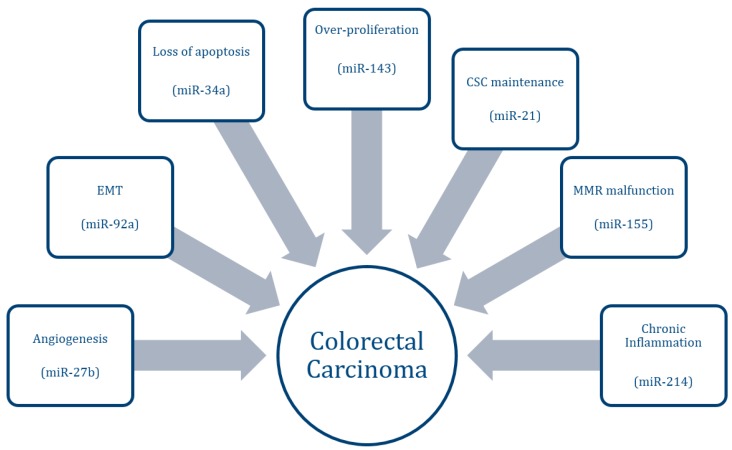
Pathways associated with colorectal cancer progression and examples of associated MicroRNAs (miRNAs).

The status of CRC in a patient spans the spectrum from benign adenoma or polyp to malignant carcinoma. This disease progression involves several mechanisms, the most notably being over-proliferation, loss of apoptotic regulation, acquisition of an invasive phenotype, increased angiogenesis, and maintenance of CSCs (Figure 1). This progression typically involves upregulation of numerous oncogenes, as well as downregulation of important tumor suppressor genes. The first association between miRNAs and CRC was identified by Michael *et al.* in 2003, who found decreased levels of miR-143 and miR-145 in CRC tissue compared to healthy tissue [6]. miRNAs may take on an oncogenic or tumor-suppressive role in their regulation of pathways leading to cancer formation. Oncogenic miRNAs, termed oncomiRs, typically target and downregulate endogenous tumor-suppressor genes. Tumor-suppressive miRNAs, on the other hand, play an important role in downregulating genes associated with growth and metastasis. The upregulation of oncomiRs and the downregulation of tumor-suppressive miRNAs therefore have profound effects on the development of cancer. An overview of current miRNAs associated with CRC is depicted in Table 1, Table 2 and Table 3.

**Table 1:** Oncogenic miRNAs in CRC.

miRNA	Target(s)	Role in Cancer	Reference
miR-21	PDCD4, TIAM1, SPRY2, PTEN, TGFBR2, CDC25A	Proliferation, Apoptosis, Invasion, Migration, CSC maintenance	[14,15,16,17,18]
miR-92a	PTEN	Proliferation, Invasion, EMT	[19]
miR-96	TP53INP1, FOXO1, FOXO3A	Proliferation	[20]
miR-135a	APC	Proliferation	[21]
miR-135b	APC	Proliferation	[21]
miR-155	MLH1, MSH2, MSH6	DNA damage response	[22]
miR-214	PTEN, PDLIM2	Inflammation	[23]
miR-224	SMAD4	Metastasis	[24]

PDCD4: programmed Cell Death 4; TIAM1: T-Cell Lymphoma Invasion And Metastasis 1; SPRY2: Sprouty Homolog 2; PTEN: Phosphatase And Tensin Homolog; TGFBR2: Transforming Growth Factor, Beta Receptor II; CDC25A: Cell Division Cycle 25A; TP53INP1: Tumor Protein P53 Inducible Nuclear Protein 1; FOXO1: Forkhead Box O1; FOXO3A: Forkhead Box O3; APC: Adenomatous Polyposis Coli; MLH1: MutL Homolog 1; MSH2: MutS Homolog 2; MSH6: MutS Homolog 6; PDLIM2: PDZ And LIM Domain 2; SMAD4: SMAD Family Member 4.

**Table 2:** Tumor-suppressive miRNAs in CRC.

miRNA	Target(s)	Role in Cancer	Reference
let-7	KRAS	Proliferation	[25]
miR-7	EGFR, RAF1	Proliferation	[26]
miR-18a*	KRAS	Proliferation	[27]
miR-26b	TAF12, PTP4A1, CHFR, ALS2CR2	Proliferation, Apoptosis, Invasion, Migration	[28]
miR-27b	VEGFC	Proliferation, Angiogenesis	[12]
miR-34a	SIRT1	Apoptosis	[29]
miR-101	SPHK1	Angiogenesis	[30]
miR-126	VEGFA	Angiogenesis	[31]
miR-143	KRAS, IGF1R	Proliferation	[32,33]
miR-144	MTOR	Proliferation	[34]
miR-145	IRS1, NRAS, IGF1R	Proliferation, Invasion, Migration, Angiogenesis	[33,35]
miR-194	AKT2	Proliferation, Apoptosis, Invasion, Migration	[36]
miR-195	BCL2	Apoptosis	[37]
miR-320a	CTNNB1	Proliferation	[38]
miR-365	BCL2, CCND1	Apoptosis	[39]
miR-491	BCLXL	Apoptosis	[40]

KRAS: Kirsten Rat Sarcoma Viral Oncogene Homolog; EGFR: Epidermal Growth Factor Receptor; RAF1: Raf-1 Proto-Oncogene; TAF12: TAF12 RNA Polymerase II, TATA Box Binding Protein (TBP)-Associated Factor, 20 kDa; PTP4A1: Protein Tyrosine Phosphatase Type IVA, Member 1; CHFR: Checkpoint With Forkhead And Ring Finger Domains; ALS2CR2: STE20-Related Kinase Adaptor Beta; VEGFC: Vascular Endothelial Growth Factor C; SIRT1: Sirtuin 1; SPHK1: Sphingosine Kinase 1; VEGFA: Vascular Endothelial Growth Factor A; IGF1R: Insulin-Like Growth Factor 1 Receptor; MTOR: Mechanistic Target Of Rapamycin; IRS1: Insulin Receptor Substrate 1; NRAS: Neuroblastoma RAS Viral (V-Ras) Oncogene Homolog; AKT2: V-Akt Murine Thymoma Viral Oncogene Homolog 2; BCL2: B-Cell CLL/Lymphoma 2; CTNNB1: Catenin (Cadherin-Associated Protein), Beta 1, 88kDa; CCND1: Cyclin D1; BCLXL: BCL2-Like 1.

**Table 3:** miRNAs associated with chemotherapy resistance/sensitization.

miR-17-5p	PTEN	Chemotherapy resistance, Migration	[41]
miR-140	HDAC4	Chemotherapy resistance, Proliferation, CSC maintenance	[42]
miR-192	TYMS	Chemotherapy resistance, Proliferation	[43]
miR-203	ATM	Chemotherapy resistance	[44]
miR-215	TYMS	Chemotherapy resistance, Proliferation	[43]
miR-222	ADAM17	Chemotherapy sensitization, Apoptosis	[45]
miR-506	PPARA	Chemotherapy resistance	[46]

PTEN: Phosphatase And Tensin Homolog; HDAC4: Histone Deacetylase 4; TYMS: Thymidylate Synthetase; ATM: Ataxia Telangiectasia Mutated; ADAM17: ADAM Metallopeptidase Domain 17; PPARA: Peroxisome Proliferator-Activated Receptor Alpha.

Uncontrolled growth and proliferation are key components to the development of a primary tumor. A major pathway associated with uncontrolled proliferation in CRC is the MAP kinase pathway, involving proteins such as RAS, RAF, and ERK. Several miRNAs have been shown to regulate proteins involved in this pathway. Let 7, miR-143, miR-18a* and miR-145, for example, downregulate RAS and act as tumor-suppressive miRNAs in CRC [25,27,32,35]. The PI3K pathway is another important signaling pathway associated with cell cycle regulation. Dysfunction of its associated proteins, such as AKT and mTOR, is a key step in the development of colorectal tumorigenesis. miR-194, as one example, has been found to repress AKT2 expression, decrease the activity of the PI3K pathway, and in turn hinder proliferation [36]. miR-144 has also been found to inhibit this pathway through negative regulation of mTOR [34]. Receptor tyrosine kinases (RTKs) play a vital role in activating the signaling pathways involved in CRC development. miR-143 and miR-145 are two miRNAs shown to have anti-tumor effects in CRC through their downregulation of insulin-like growth factor 1 receptor (*IGF1R*) [33].

A necessary characteristic for uncontrolled cell growth involves the loss of appropriate apoptotic control. Several miRNAs have been shown to be involved, and dysregulated, in apoptotic regulation for CRC. miRNAs miR-195 and miR-491, for instance, have been found to promote apoptosis in CRC cells through the targeting of B-Cell CLL/Lymphoma 2 (*BCL2*) and BCL2-Like 1 (*BCLXL*), respectively [37,40]. Mutations in *P53*, a tumor-suppressor gene that is important in cell cycle regulation and apoptosis, are common in CRC. miRNAs can negatively or positively influence the activity of this protein. miR-96, for example, is found upregulated in CRC and is shown to target p53 inducible nuclear protein 1 (*TP53INP1*), exerting downregulatory effects on p53 activity [20]. miR-34a, on the other hand, has been found to increase p53 activity through its inhibition of Sirtuin-1 (*SIRT1*) [29].

The development of certain types of CRC is also closely related to dysfunctions in mismatch repair (MMR) enzymes. These enzymes act to repair mistakes in the genome brought about by DNA synthesis. Dysfunctions in this process can unsurprisingly lead to mutations in protein-coding genes, resulting in pathology, as well as introducing instability in repetitive stretches of DNA termed microsatellites. Microsatellite instability (MSI) is a hallmark feature in defective MMR. Hereditary non-polyposis colorectal cancer (HNPCC) is the most frequent hereditary form of CRC, and is caused by mutations in MMR enzymes [47]. In addition, around 15% of sporadic CRC cases present with a malfunctioning MMR process [47]. miRNAs have recently been shown to regulate genes associated with MMR in CRC. miR-155, for example, targets *hMLH1*, *hMSH2*, and *hMSH6*, pivotal genes involved in this process [22].

The acquisition of an invasive and migratory phenotype by cancer cells is a necessary step in the development of metastasis. miR-21, which has been described as one of the most highly upregulated miRNAs in CRC, downregulates several genes involved in controlling invasion and migration, including *PDCD4*, *TIAM1*, *SPRTY*, and *PTEN* [15,16,17,18,48]. miR-224 has also recently been found to enhance the metastasis of CRC through its modulation of *SMAD4* [24]. EMT is an important step associated with the development of an invasive phenotype, and miR-92a has been shown to promote this process through the suppression of E-cadherin [19]. Angiogenesis is another key component of metastasis, and miR-27b has shown anti-angiogenic effects through its targeting of vascular endothelial growth factor C (*VEGFC*) [12].

Alterations in the WNT signaling pathway are commonly found dysregulated in the initiation and progression of CRC. Adenomatous polyposis coli (*APC*) inhibits WNT signaling via its sequestration of beta-catenin, and has been implicated as one of the most commonly downregulated genes in CRC [49]. The hereditary loss of this tumor-suppressor is characteristically seen in Familial Adenomatous Polyposis (FAP), a genetic disorder that substantially increases the risk for the development of CRC. Inactivation of *APC* in colon adenocarcinoma cells has been found associated with increased levels of miR-135a/b [21]. In addition, miR-320a has been shown to directly target beta-catenin, thereby decreasing the oncogenic capabilities of this pathway [38]. WNT signaling has also shown close associations with the presence of CSCs in CRC [38]. Cancer stem cells have the ability to repopulate a tumor population after treatment, and are vitally important with regards to recurrence and therapeutic resistance. miR-21 has been shown to be critically involved in the maintenance of CSC in CRC cells [14].

Chronic inflammatory disorders of the colon, such as ulcerative colitis and Crohn’s disease, have shown to pose a greater risk for the development of CRC. The genetic alterations behind this association play a pivotal role in the ultimate development of cancer. While the “classical” CRC schema progresses from an adenoma to carcinoma, UC-CRC progresses through an intermediate dysplasia stage. This unique development may culminate in cancer via several mechanisms, and miRNAs have been shown to influence a number of these pathways. For example, miR-214 has been shown to be increased in CRC associated with colitis, while no significant differences were found in sporadic CRC and control specimens. In addition, miR-214 has been shown to indirectly activate NF-κB, a potent pro-inflammatory molecule, through direct repression of *PTEN* and PDZ and LIM domain2 (*PDLIM2*). This lends support for the role of miR-214 in the progression of CRC specifically from colitis [23]. Researches suggest that miRNAs could be used to stratify the progression of UC-CRC from UC. For instance, miR-375 shows a significant differential expression between UC patients and UC-CRC patients [50]. In another study, a panel of five miRNAs (miR-193b, miR-373, let-7e, miR-15b, miR-372) were shown to be differentially expressed in the development of UC-CRC from UC and Crohn’s disease [51]. The level of miRNA could also be used to differentiate between UC-CRC and sporadic CRC. Benderska *et al*. have found increased levels of miR-26b in the tissues of two cohorts of patients with UC and UC-CRC, while decreased levels of miR-26b are typically associated with sporadic CRC. It was further demonstrated that miR-26b likely contributes to the progression of UC-CRC [52].

## 3. Diagnostic Potential of miRNAs in CRC

The five-year survival rate drops from around 90% for early-stage CRC patients to less than 15% for those with metastatic disease [4]. Early detection methods of screening and diagnosis are therefore paramount to decreasing the mortality caused by CRC. Screening and diagnosis of CRC, however, remains a significant challenge in today’s healthcare. According to the American College of Gastroenterology, the currently preferred method of screening is colonoscopy [53]. Burdened by an invasive, costly, uncomfortable procedure requiring significant bowel and dietary restrictions, this method is not desirable for patients. The less invasive screening methods, such as fecal occult blood test (FOBT), have also been found to cause discomfort and embarrassment among patients, as well as having a suboptimal sensitivity [54,55]. The compliance rate for CRC screening in 2012 was found to be around 65%, detailing the need for improved techniques [56]. The development of a non-invasive, affordable, and highly specific and sensitive test would certainly improve the current status of CRC detection.

In the past several years, miRNAs have been found to show incredible promise in offering improved screening and diagnostic methods for both primary and metastatic CRC tumors. Firstly, the utility of miRNAs eliminates many of the negative characteristics associated with currently used methods. The use of miRNAs in screening and diagnosis avoids the discomfort and invasiveness that is typically associated with colonoscopy. In addition, dietary restrictions and stool preparations are not warranted for plasma miRNAs, as they are with FOBT. Secondly, miRNAs show tissue specific expression and are relatively stable, indicating their potential advantage as biomarkers [57,58]. Differential expression of miRNAs has been able to discriminate colon cancers from rectal cancers, as well as MSI CRC from microsatellite stable (MSS) CRC [59,60]. miR-92a has been shown to have a high sensitivity in the differentiation of proximal and distal CRC, and elevated miR-92a levels have been able to distinguish CRC from inflammatory bowel disease (IBD) and gastric cancer (GC) [55,61]. Thirdly, miRNA expression profiles have been found to be highly accurate in predicting CRC [58]. Upregulated plasma levels of miR-92a, for example, have been shown to have a high specificity (70%) and sensitivity (89%) in predicting CRC [55]. A separate study corroborated these results, and found that miR-29a is also able to distinguish between CRC and healthy controls with a high specificity (89.1%) and sensitivity (69%) [62].

miRNAs have also shown to remain at high levels in stool after 72 h, and fecal screening methods are very reproducible [61]. Koga *et al.* have found that fecal miR-106a was able to predict CRC after a false-negative prediction by FOBT [63]. Furthermore, fecal miR-221 has shown to be more specific (74%) at predicting CRC than plasma levels of miR-221 (41%) [64].

Single miRNA expression levels are sometimes presented with conflicting results, and combinations of miRNA levels have shown to be better predictors of CRC [65]. The combined use of miR-92a and miR-29a yielded an improved specificity and sensitivity for detection of advanced adenomas and CRC [62]. Yau *et al.* also found that the sensitivity and specificity of the fecal miR-221 and miR-18a signature were higher in predicting CRC than the use of either miRNA independently [64].

Metastasis plays a prominent role in mortality due to CRC. As stated previously, miRNAs play an important role in the development of metastatic features of tumors, and it follows that these signatures can also serve as diagnostic biomarkers. Hur.*et al.* have found a miRNA expression profile consisting of let-7i, miR-10b, and miR-885-5p that is specific for CRC metastasis [66]. miR-592 and 552 levels could also distinguish between primary lung cancer and lung metastasis secondary to CRC [67]. Carcinoembryonic antigen (CEA) is a currently used biomarker for CRC metastasis, and Cheng *et al.* have shown that miR-141 may be used in conjunction with CEA to increase its predictive capabilities [68,69].

## 4. Prognostic Implications of miRNAs in CRC

In addition to their potential uses in screening and diagnosis, miRNAs have shown promise as valuable markers of CRC, being able to predict recurrence, survival, and drug response.

Post-surgical recurrence occurs in about 45% of CRC patients, resulting in a worsened prognosis [70]. The ability to accurately predict recurrence risk would be very beneficial in the decision to treat a patient with adjuvant therapy after surgical resection. It has been found that certain miRNAs may serve as better prognostic indicators than currently used factors. For example, miR-200c was found to be a better predictor of relapse in stage II and III CRC patients than either CEA levels or pathological staging [71].

Overall survival (OS) and progression-free survival (PFS) often go hand in hand with relapse. As expected, miRNAs can provide valuable information with regards to this aspect of CRC as well. It has been found, for example, that increased levels of miR-126 are significantly associated with a longer PFS in CRC patients [72]. Certain polymorphisms within miRNAs have also been shown to serve as potential prognostic markers. For example, Xing *et al.* found single-nucleotide polymorphisms (SNPs) in pre-miR-423 and pre-miR-608 that were significant predictors of OS and PFS in CRC [73].

Perhaps one of the most important prognostic applications of miRNAs in CRC is their ability to predict a therapeutic response to certain chemotherapy agents. Chemotherapy remains a primary treatment option for patients with CRC, and the development of resistance remains a significant obstacle. Chemotherapy resistance is often multifactorial, and may involve maintenance of CSCs, increased ability to repair DNA damage, and reduced apoptosis. miRNAs are intimately involved in these processes and therefore help to modulate the development of resistance.

Most chemotherapeutic regimens for CRC consist of a combination of several different agents. One commonly used regimen is composed of oxaliplatin, 5-fluorouracil (5-FU), and leucovorin (FOLFOX) [74]. Several miRNAs have been shown to act as markers that have the ability to predict a patient's response to these agents. This predictive information would help prevent unnecessary treatment for some patients who otherwise would not benefit, as well as providing a better outcome for those patients who are predicted to be responsive. miR-625-3p and miR-203 for example, were found to be upregulated in CRC cells resistant to oxaliplatin [44,75]. Upregulation of miR-17-5p has been found to be associated with resistance to 5-FU in CRC, possibly through its repression of *PTEN* [41]. Other miRNAs, such as miR-192 and miR-215, have been shown to target thymidylate synthase (*TYMS*), a potential target of 5-FU. Surprisingly, however, these miRNAs seemed to increase resistance to 5-FU, rather than improving the drug’s efficacy [43]. This is thought to be due to the complex actions of miR-192 and miR-215. Polymorphisms within miRNAs have also shown prognostic value in determining drug response. For example, variants in miR-291-1, miR-608, miR-26a-1, and miR-100 have shown associations with response to chemotherapy treatment [76,77].

The effect of certain miRNAs on chemotherapy resistance has also been related to the patient’s p53 status. In patients with a mutated p53, it was shown that inhibition of miR-520g resulted in an improved sensitivity to 5-FU [78]. It was also found by Song *et al.* that chemoresistance due to an upregulation of miR-140 in CRC cells following methotrexate treatment was dependent on a functional p53 protein [42].

Anti-EGFR monoclonal antibodies, such as cetuximab, belong to another class of therapeutic agents utilized for CRC treatment [79]. It is widely known that patients with a mutated *KRAS* gene have a poor response to these treatments, and considering the strong association between miRNAs and KRAS signaling, it is not surprising that certain miRNAs have prognostic value for predicting their therapeutic response. It has been found that low levels of miR-181a, for example, may be associated with a worse prognosis in patients treated with anti-EGFR therapy [79]. miR-7 and the miR-99a/let-7c/miR-125b signature may also play an important role in regulating CRC patient responses to anti-EGFR therapies [26,80].

Additional classes of chemotherapeutic drugs have also shown differing sensitivities to CRC cells due to altered levels of miRNAs. Upregulation of miR-101, for example, has been shown to increase the sensitivity of CRC cells to microtubule inhibitors, such as paclitaxel, and anthracyclines, such as doxorubicin [30]. Tong *et al.* have also shown a correlation between hydroxycamptothecin resistance, which is a topoisomerase I inhibitor utilized in the drug irinotecan, and upregulated miR-506 expression [46]. In addition, miR-144 has shown promise in predicting CRC cell susceptibility to rapamycin [34].

Radiation therapy has been shown to be an effective adjuvant therapy in patients with rectal cancer [81]. Similar to chemotherapy, radiotherapy sensitivity has been shown to be associated with miRNA levels. Yang *et al*., for example, have recently found an association between levels of miR-100 and the development of radioresistance in CRC cells [82].

## 5. Targeting miRNAs for CRC Therapy

The intimate involvement miRNAs have in the pathophysiology of CRC lends significant support for their utilization in novel therapeutics. As mentioned previously, several currently used chemotherapeutic drugs show an altered efficacy depending on the expression levels of certain miRNAs. It has been suggested that this knowledge can be utilized through introduction of miRNA mimetics or inhibitors to improve drug response. For example, miR-143 has been found to increase CRC cell sensitivity to 5-FU [83]. It is therefore plausible that a combined therapy of 5-FU and a miR-143 mimetic would increase a patient's positive prognosis. Functional studies have also demonstrated that induction of miR-365 in CRC cells enhanced apoptosis mediated by 5-FU [39]. Furthermore, it has been found that the downregulation of chemoresistant genes, such as *SIRT-1*, can be achieved through upregulation of their regulator miRNAs, such as miR-34a, resulting in an improved drug response [31].

Several miRNAs have also shown promise in combination therapy with anti-EGFR CRC therapeutics. miR-147, for example, has been shown to increase CRC sensitivity to gefitinib after induction of mesenchymal-epithelial transition (MET) [84]. While anti-EGFR treatment is not effective in patients with a mutated KRAS protein, Ruzzo *et al*. found that upregulation of let-7a may rescue anti-EGFR sensitivity in such CRC patients [85].

With regards to additional classes of chemotherapeutics, miR-192 has shown promise as a sensitizer of CRC cells to methotrexate. Due to its pleomorphic effects, the combination of miR-192 with methotrexate has shown greater efficacy than alternative combinatorial treatments [86]. Furthermore, ADAM-17, a protein potentially involved in multi-drug resistance (MDR) in CRC cells, is regulated by miR-222, lending support for its utilization in combination therapy for CRC [45].

It is plausible to consider that the restoration of downregulated tumor-suppressor miRNAs in CRC patients may provide a significant therapeutic benefit. Many miRNAs have shown anti-tumorigenic effects, and it would seem appropriate to base potential therapies on miRNAs involved in multiple tumorigenic pathways. miR-26b, for example, has been shown to target several different oncogenic genes in CRC (Table 2). Furthermore, research in xenograft models suggests that miR-26b mimics are non-toxic and may serve as potential treatments in humans [28]. miR-21, on the other hand, has been described as a powerful oncogenic miRNA, contributing to carcinogenesis via several different mechanisms (Table 1). Downregulation of miR-21 has been suggested as a therapeutic target based on its widespread functional associations with CRC.

miRNAs have also shown promise in the development of drugs targeting specific novel pathways associated with cancer. Anti-angiogenic therapies, for example, are being used more frequently in CRC treatment. Bevacizumab is one currently approved monoclonal antibody that has anti-angiogenic effects by targeting VEGF [87]. miR-126 has also shown therapeutic potential in CRC through its targeting of *VEGFA* and its subsequent regulation of angiogenesis [31].

In attempting to downregulate an oncogenic or drug resistance-promoting miRNA, one would typically use small molecule inhibitors of the target miRNA, termed antagomiRs [88]. These agents act through high affinity binding to the target miRNA strand, resulting in its degradation or repression. One potential complication with antagomiR use, however, is their ability to bind to unintended RNAs, causing unwanted side effects [88].

miRNA replacement therapy, on the other hand, attempts to increase the effects of tumor-suppressive and drug sensitizing miRNAs through introduction of analogous miRNA molecules. This can be done through a gene replacement therapy similar to that utilized for small interfering RNAs (siRNAs), which is thought to present fewer difficulties than gene therapy with protein-coding genes. Innovative delivery methods utilizing liposomal and polymerase-based techniques are currently being explored. It is also believed that these mimics will produce fewer negative side effects than typical gene replacement treatment. Current therapies utilizing miR-34a and let-7 mimics, both of which have tumor-suppressor roles in CRC, have shown therapeutic efficacy in mouse models of lung and prostate cancers [88]. Excitingly, the first clinical trial utilizing miRNAs in cancer treatment began in 2013 with MRX34, a liposomal-based mimic of miR-34 to be used in patients with advanced hepatocellular carcinoma [89]. While the theoretical applications of miRNA therapy in CRC is very promising, its delivery and practical applications still present significant challenges. Suboptimal pharmacokinetic properties represent the need for increased stability, improved uptake, and more efficient clearance for these novel drugs [88].

## 6. Challenges and Obstacles

While research suggests that the use of miRNAs as potential biomarkers in CRC is promising, there are considerable challenges hindering the clinical application of miRNAs for CRC diagnostic or prognostic purposes. The practical way of using miRNAs as clinically relevant biomarkers for CRC is to measure miRNA levels in stool or in body fluid such as plasma. However, technically this can be very challenging, largely due to lack of standardization in the experimental approaches. For instance, plasma collection time point, plasma collection procedures, storage conditions, transportation methods, detection method, and normalizations could all affect the final miRNA detection and analysis. This is further complicated by recent findings of enrichment of miRNAs in exosomes, which can be separated from the serum by using ultracentrifugation methods. Therefore, different protocols used in the plasma RNA extraction will inevitably lead to discrepancies in the research findings by different groups. Standard operation procedures from sample collection to final analysis, which are still lacking right now, are key for successful translation of miRNA discovery into clinical settings. In addition, oligonucleotide-based miRNA therapeutics need to overcome challenges related to uptake efficiency, tissue and tumor specificity, unintended side effects, and a more effective and safe delivery system.

Another obstacle impeding the application of miRNA knowledge into clinical use lies within the inherent characteristics of miRNA molecules. The expression and function of miRNAs are fine-tuned, and largely dependent on the cellular context. One miRNA could have multiple targets, but these targets may not all be regulated by the miRNA in every circumstance. How a miRNA targets one target but not another in a specific context remains to be discovered. Until these questions can be clearly answered, utilizing miRNAs as biological functional surrogates will be ambiguous, and seriously hinder their use as clinical biomarkers and therapeutics.

## 7. Conclusions

Since the initial discovery of their associations with cancer in 2002, the amount of research and knowledge involving miRNAs has exploded. While the clinical use of miRNAs still faces many challenges, we believe this area of research holds significant implications with regards to improvements in CRC morbidity and mortality. CRC is characterized by genetic alterations, and miRNAs have proven vital in the regulation of genetic expression. As this paper has attempted to convey, an enormous amount of information detailing the specific roles miRNAs play in CRC pathology currently exists, and this knowledge base continues to expand. We believe that with improved understanding of miRNA biology, delivery system innovations, and detection methodologies, we will see the utility of miRNA use in the diagnosis and treatment of CRC patients in the near future. Harnessing this current and future information can potentially change the way CRC is diagnosed and treated, improving the livelihood of millions of people worldwide.

## References

[1] Haggar F.A., Boushey R.P. (2009). Colorectal cancer epidemiology: Incidence, mortality, survival, and risk factors. Clin. Colon Rectal Surg..

[2] Siegel R., Desantis C., Jemal A. (2014). Colorectal cancer statistics, 2014. CA Cancer J. Clin..

[3] Edwards B.K., Ward E., Kohler B.A., Eheman C., Zauber A.G., Anderson R.N., Jemal A., Schymura M.J., Lansdorp-Vogelaar I., Seeff L.C. (2010). Annual report to the nation on the status of cancer, 1975–2006, featuring colorectal cancer trends and impact of interventions (risk factors, screening, and treatment) to reduce future rates. Cancer.

[4] Howlader N., Noone A.M., Krapcho M., Garshell J., Miller D., Altekruse S.F., Kosary C.L., Yu M., Ruhl J., Tatalovich Z. (2015). SEER Cancer Statistics Review, 1975–2012.

[5] Calin G.A., Croce C.M. (2006). MicroRNA signatures in human cancers. Nat. Rev. Cancer.

[6] Michael M.Z., O’Connor S.M., van Holst Pellekaan N.G., Young G.P., James R.J. (2003). Reduced accumulation of specific microRNAs in colorectal neoplasia. Mol. Cancer Res..

[7] Pichler M., Calin G.A. (2015). MicroRNAs in cancer: From developmental genes in worms to their clinical application in patients. Br. J. Cancer.

[8] Ha M., Kim V.N. (2014). Regulation of microRNA biogenesis. Nat. Rev. Mol. Cell Biol..

[9] Feig J.L., Giles K.M., Osman I., Franks A.G. (2015). How microRNAs modify protein production. J. Investig. Dermatol..

[10] Calin G.A., Dumitru C.D., Shimizu M., Bichi R., Zupo S., Noch E., Aldler H., Rattan S., Keating M., Rai K. (2002). Frequent deletions and down-regulation of micro-RNA genes miR15 and miR16 at 13q14 in chronic lymphocytic leukemia. Proc. Natl. Acad. Sci. USA.

[11] Garzon R., Calin G.A., Croce C.M. (2009). MicroRNAs in Cancer. Annu. Rev. Med..

[12] Ye J., Wu X., Wu D., Wu P., Ni C., Zhang Z., Chen Z., Qiu F., Xu J., Huang J. (2013). miRNA-27b targets vascular endothelial growth factor C to inhibit tumor progression and angiogenesis in colorectal cancer. PLoS ONE.

[13] Hur K., Toiyama Y., Takahashi M., Balaguer F., Nagasaka T., Koike J., Hemmi H., Koi M., Boland C.R., Goel A. (2013). MicroRNA-200c modulates epithelial-to-mesenchymal transition (EMT) in human colorectal cancer metastasis. Gut.

[14] Yu Y., Kanwar S.S., Patel B.B., Oh P.S., Nautiyal J., Sarkar F.H., Majumdar A.P. (2012). MicroRNA-21 induces stemness by downregulating transforming growth factor beta receptor 2 (TGFβR2) in colon cancer cells. Carcinogenesis.

[15] Asangani I.A., Rasheed S.A., Nikolova D.A., Leupold J.H., Colburn N.H., Post S., Allgayer H. (2008). MicroRNA-21 (miR-21) post-transcriptionally downregulates tumor suppressor Pdcd4 and stimulates invasion, intravasation and metastasis in colorectal cancer. Oncogene.

[16] Cottonham C.L., Kaneko S., Xu L. (2010). miR-21 and miR-31 converge on TIAM1 to regulate migration and invasion of colon carcinoma cells. J. Biol. Chem..

[17] Sayed D., Rane S., Lypowy J., He M., Chen I.Y., Vashistha H., Yan L., Malhotra A., Vatner D., Abdellatif M. (2008). MicroRNA-21 targets Sprouty 2 and promotes cellular outgrowths. Mol. Biol. Cell.

[18] Xiong B., Cheng Y., Ma L., Zhang C. (2013). miR-21 regulates biological behavior through the PTEN/PI-3 K/Akt signaling pathway in human colorectal cancer cells. Int. J. Oncol..

[19] Zhang G., Zhou H., Xiao H., Liu Z., Tian H., Zhou T. (2014). MicroRNA-92a functions as an oncogene in colorectal cancer by targeting PTEN. Dig. Dis. Sci..

[20] Gao F., Wang W. (2015). MicroRNA-96 promotes the proliferation of colorectal cancer cells and targets tumor protein p53 inducible nuclear protein 1, forkhead box protein O1 (FOXO1) and FOXO3a. Mol. Med. Rep..

[21] Nagel R., le Sage C., Diosdado B., van der Waal M., Oude Vrielink J.A., Bolijn A., Meijer G.A., Agami R. (2008). Regulation of the adenomatous polyposis coli gene by the miR-135 family in colorectal cancer. Cancer Res..

[22] Valeri N., Gasparini P., Fabbri M., Braconi C., Veronese A., Lovat F., Adair B., Vannini I., Fanini F., Bottoni A. (2010). Modulation of mismatch repair and genomic stability by miR-155. Proc. Natl. Acad. Sci. USA.

[23] Polytarchou C., Hommes D.W., Palumbo T., Hatziapostolou M., Koutsioumpa M., Koukos G., van der Meulen-de Jong A.E., Oikonomopoulos A., van Deen W.K., Vorvis C. (2015). MicroRNA214 is associated with progression of ulcerative colitis, and inhibition reduces development of colitis and colitis-associated cancer in mice. Gastroenterology.

[24] Ling H., Pickard K., Ivan C., Isella C., Ikuo M., Mitter R., Spizzo R., Bullock M.D., Braicu C., Pileczki V. (2015). The clinical and biological significance of miR-224 expression in colorectal cancer metastasis. Gut.

[25] Johnson S.M., Grosshans H., Shingara J., Byrom M., Jarvis R., Cheng A., Labourier E., Reinert K.L., Brown D., Slack F.J. (2005). RAS is regulated by the let-7 microRNA family. Cell.

[26] Suto T., Yokobori T., Yajima R., Morita H., Fujii T., Yamaguchi S., Altan B., Tsutsumi S., Asao T., Kuwano H. (2015). MicroRNA-7 expression in colorectal cancer is associated with poor prognosis and regulates cetuximab sensitivity via EGFR regulation. Carcinogenesis.

[27] Tsang W.P., Kwok T.T. (2009). The miR-18a* microRNA functions as a potential tumor suppressor by targeting on K-Ras. Carcinogenesis.

[28] Ma Y.L., Zhang P., Wang F., Moyer M.P., Yang J.J., Liu Z.H., Peng J.Y., Chen H.Q., Zhou Y.K., Liu W.J. (2011). Human embryonic stem cells and metastatic colorectal cancer cells shared the common endogenous human microRNA-26b. J. Cell. Mol. Med..

[29] Yamakuchi M., Ferlito M., Lowenstein C.J. (2008). miR-34a repression of SIRT1 regulates apoptosis. Proc. Natl. Acad. Sci. USA.

[30] Chen M.B., Yang L., Lu P.H., Fu X.L., Zhang Y., Zhu Y.Q., Tian Y. (2015). MicroRNA-101 down-regulates sphingosine kinase 1 in colorectal cancer cells. Biochem. Biophys. Res. Commun..

[31] Stiegelbauer V., Perakis S., Deutsch A., Ling H., Gerger A., Pichler M. (2014). MicroRNAs as novel predictive biomarkers and therapeutic targets in colorectal cancer. World J. Gastroenterol..

[32] Chen X., Guo X., Zhang H., Xiang Y., Chen J., Yin Y., Cai X., Wang K., Wang G., Ba Y. (2009). Role of miR-143 targeting KRAS in colorectal tumorigenesis. Oncogene.

[33] Su J., Liang H., Yao W., Wang N., Zhang S., Yan X., Feng H., Pang W., Wang Y., Wang X. (2014). miR-143 and miR-145 regulate IGF1R to suppress cell proliferation in colorectal cancer. PLoS ONE.

[34] Iwaya T., Yokobori T., Nishida N., Kogo R., Sudo T., Tanaka F., Shibata K., Sawada G., Takahashi Y., Ishibashi M. (2012). Downregulation of miR-144 is associated with colorectal cancer progression via activation of mTOR signaling pathway. Carcinogenesis.

[35] Yin Y., Yan Z.P., Lu N.N., Xu Q., He J., Qian X., Yu J., Guan X., Jiang B.H., Liu L.Z. (2013). Downregulation of miR-145 associated with cancer progression and VEGF transcriptional activation by targeting N-RAS and IRS1. Biochim. Biophys. Acta.

[36] Zhao H.J., Ren L.L., Wang Z.H., Sun T.T., Yu Y.N., Wang Y.C., Yan T.T., Zou W., He J., Zhang Y. (2014). miR-194 deregulation contributes to colorectal carcinogenesis via targeting AKT2 pathway. Theranostics.

[37] Liu L., Chen L., Xu Y., Li R., Du X. (2010). MicroRNA-195 promotes apoptosis and suppresses tumorigenicity of human colorectal cancer cells. Biochem. Biophys. Res. Commun..

[38] Sun J.Y., Huang Y., Li J.P., Zhang X., Wang L., Meng Y.L., Yan B., Bian Y.Q., Zhao J., Wang W.Z. (2012). MicroRNA-320a suppresses human colon cancer cell proliferation by directly targeting β-catenin. Biochem. Biophys. Res. Commun..

[39] Nie J., Liu L., Zheng W., Chen L., Wu X., Xu Y., Du X., Han W. (2012). MicroRNA-365, down-regulated in colon cancer, inhibits cell cycle progression and promotes apoptosis of colon cancer cells by probably targeting Cyclin D1 and Bcl-2. Carcinogenesis.

[40] Nakano H., Miyazawa T., Kinoshita K., Yamada Y., Yoshida T. (2010). Functional screening identifies a microRNA, miR-491 that induces apoptosis by targeting BCL-X_L_ in colorectal cancer cells. Int. J. Cancer.

[41] Fang L., Li H., Wang L., Hu J., Jin T., Wang J., Yang B.B. (2014). MicroRNA-17-5p promotes chemotherapeutic drug resistance and tumour metastasis of colorectal cancer by repressing PTEN expression. Oncotarget.

[42] Song B., Wang Y., Xi Y., Kudo K., Bruheim S., Botchkina G.I., Gavin E., Wan Y., Formentini A., Kornmann M. (2009). Mechanism of chemoresistance mediated by miR-140 in human osteosarcoma and colon cancer cells. Oncogene.

[43] Boni V., Bitarte N., Cristobal I., Zarate R., Rodriguez J., Maiello E., Garcia-Foncillas J., Bandres E. (2010). miR-192/miR-215 influence 5-fluorouracil resistance through cell cycle-mediated mechanisms complementary to its post-transcriptional thymidilate synthase regulation. Mol. Cancer Ther..

[44] Zhou Y., Wan G., Spizzo R., Ivan C., Mathur R., Hu X., Ye X., Lu J., Fan F., Xia L. (2014). miR-203 induces oxaliplatin resistance in colorectal cancer cells by negatively regulating ATM kinase. Mol. Oncol..

[45] Xu K., Liang X., Shen K., Sun L., Cui D., Zhao Y., Tian J., Ni L., Liu J. (2012). miR-222 modulates multidrug resistance in human colorectal carcinoma by down-regulating ADAM-17. Exp. Cell Res..

[46] Tong J.L., Zhang C.P., Nie F., Xu X.T., Zhu M.M., Xiao S.D., Ran Z.H. (2011). MicroRNA 506 regulates expression of PPAR alpha in hydroxycamptothecin-resistant human colon cancer cells. FEBS Lett..

[47] Colling R., Church D.N., Carmichael J., Murphy L., East J., Risby P., Kerr R., Chetty R., Wang L.M. (2015). Screening for Lynch syndrome and referral to clinical genetics by selective mismatch repair protein immunohistochemistry testing: An audit and cost analysis. J. Clin. Pathol..

[48] De Krijger I., Mekenkamp L.J., Punt C.J., Nagtegaal I.D. (2011). MicroRNAs in colorectal cancer metastasis. J. Pathol..

[49] De Sousa E.M., Vermeulen L., Richel D., Medema J.P. (2011). Targeting Wnt signaling in colon cancer stem cells. Clin. Cancer Res..

[50] Patel M., Verma A., Aslam I., Pringle H., Singh B. (2015). Novel plasma microRNA biomarkers for the identification of colitis-associated carcinoma. Lancet.

[51] Kanaan Z., Rai S.N., Eichenberger M.R., Barnes C., Dworkin A.M., Weller C., Cohen E., Roberts H., Keskey B., Petras R.E. (2012). Differential microRNA expression tracks neoplastic progression in inflammatory bowel disease-associated colorectal cancer. Hum. Mutat..

[52] Benderska N., Dittrich A.L., Knaup S., Rau T.T., Neufert C., Wach S., Fahlbusch F.B., Rauh M., Wirtz R.M., Agaimy A. (2015). miRNA-26b overexpression in ulcerative colitis-associated carcinogenesis. Inflamm. Bowel Dis..

[53] Rex D., Johnson D., Anderson J., Schoenfeld P., Burke C., Inadomi J. (2009). American college of gastroenterology guidelines for colorectal cancer screening 2008. Am. J. Gastroenterol..

[54] Gordon N.P., Green B.B. (2015). Factors associated with use and non-use of the Fecal Immunochemical Test (FIT) kit for colorectal cancer screening in response to a 2012 outreach screening program: A survey study. BMC Public Health.

[55] Ng E.K., Chong W.W., Jin H., Lam E.K., Shin V.Y., Yu J., Poon T.C., Ng S.S., Sung J.J. (2009). Differential expression of microRNAs in plasma of patients with colorectal cancer: A potential marker for colorectal cancer screening. Gut.

[56] Klabunde C., Joseph D., King J., White A., Plescia M. (2013). Vital signs: Colorectal cancer screening test use—United States, 2012. Morb. Mortal. Wkly. Rep..

[57] Mitchell P.S., Parkin R.K., Kroh E.M., Fritz B.R., Wyman S.K., Pogosova-Agadjanyan E.L., Peterson A., Noteboom J., O’Briant K.C., Allen A. (2008). Circulating microRNAs as stable blood-based markers for cancer detection. Proc. Natl. Acad. Sci. USA.

[58] Lu J., Getz G., Miska E.A., Alvarez-Saavedra E., Lamb J., Peck D., Sweet-Cordero A., Ebert B.L., Mak R.H., Ferrando A.A. (2005). MicroRNA expression profiles classify human cancers. Nature.

[59] Slattery M.L., Wolff E., Hoffman M.D., Pellatt D.F., Milash B., Wolff R.K. (2011). MicroRNAs and colon and rectal cancer: Differential expression by tumor location and subtype. Genes Chromosomes Cancer.

[60] Lanza G., Ferracin M., Gafà R., Veronese A., Spizzo R., Pichiorri F., Liu C.G., Calin G.A., Croce C.M., Negrini M. (2007). mRNA/microRNA gene expression profile in microsatellite unstable colorectal cancer. Mol. Cancer.

[61] Wu C.W., Ng S.S., Dong Y.J., Ng S.C., Leung W.W., Lee C.W., Wong Y.N., Chan F.K., Yu J., Sung J.J. (2012). Detection of miR-92a and miR-21 in stool samples as potential screening biomarkers for colorectal cancer and polyps. Gut.

[62] Huang Z., Huang D., Ni S., Peng Z., Sheng W., Du X. (2010). Plasma microRNAs are promising novel biomarkers for early detection of colorectal cancer. Int. J. Cancer.

[63] Koga Y., Yamazaki N., Yamamoto Y., Yamamoto S., Saito N., Kakugawa Y., Otake Y., Matsumoto M., Matsumura Y. (2013). Fecal miR-106a is a useful marker for colorectal cancer patients with false-negative results in immunochemical fecal occult blood test. Cancer Epidemiol. Biomark. Prev..

[64] Yau T.O., Wu C.W., Dong Y., Tang C.M., Ng S.S., Chan F.K., Sung J.J., Yu J. (2014). MicroRNA-221 and microRNA-18a identification in stool as potential biomarkers for the non-invasive diagnosis of colorectal carcinoma. Br. J. Cancer.

[65] Hofsli E., Sjursen W., Prestvik W.S., Johansen J., Rye M., Tranø G., Wasmuth H.H., Hatlevoll I., Thommesen L. (2013). Identification of serum microRNA profiles in colon cancer. Br. J. Cancer.

[66] Hur K., Toiyama Y., Schetter A.J., Okugawa Y., Harris C.C., Boland C.R., Goel A. (2015). Identification of a metastasis-specific microRNA signature in human colorectal cancer. J. Natl. Cancer Inst..

[67] Kim J., Lim N.J., Jang S.G., Kim H.K., Lee G.K. (2014). miR-592 and miR-552 can distinguish between primary lung adenocarcinoma and colorectal cancer metastases in the lung. Anticancer Res..

[68] Cheng H., Zhang L., Cogdell D.E., Zheng H., Schetter A.J., Nykter M., Harris C.C., Chen K., Hamilton S.R., Zhang W. (2011). Circulating plasma miR-141 is a novel biomarker for metastatic colon cancer and predicts poor prognosis. PLoS ONE.

[69] Kanaan Z., Roberts H., Eichenberger M.R., Billeter A., Ocheretner G., Pan J., Rai S.N., Jorden J., Williford A., Galandiuk S. (2013). A plasma microRNA panel for detection of colorectal adenomas: A step toward more precise screening for colorectal cancer. Ann. Surg..

[70] Wilhelmsen M., Kring T., Jorgensen L.N., Madsen M.R., Jess P., Bulut O., Nielsen K.T., Andersen C.L., Nielsen H.J. (2014). Determinants of recurrence after intended curative resection for colorectal cancer. Scand. J. Gastroenterol..

[71] Toiyama Y., Hur K., Tanaka K., Inoue Y., Kusunoki M., Boland C.R., Goel A. (2014). Serum miR-200c is a novel prognostic and metastasis-predictive biomarker in patients with colorectal cancer. Ann. Surg..

[72] Hansen T.F., Christensen R.D., Andersen R.F., Sørensen F.B., Johnsson A., Jakobsen A. (2013). MicroRNA-126 and epidermal growth factor-like domain 7-an angiogenic couple of importance in metastatic colorectal cancer. Results from the Nordic ACT trial. Br. J. Cancer.

[73] Xing J., Wan S., Zhou F., Qu F., Li B., Myers R.E., Fu X., Palazzo J.P., He X., Chen Z., Yang H. (2012). Genetic polymorphisms in pre-microRNA genes as prognostic markers of colorectal cancer. Cancer Epidemiol. Biomark. Prev..

[74] Compton C., Tanabe K. (2015). Pathology and Prognostic Determinants of CRC.

[75] Rasmussen M.H., Jensen N.F., Tarpgaard L.S., Qvortrup C., Rømer M.U., Stenvang J., Hansen T.P., Christensen L.L., Lindebjerg J., Hansen F. (2013). High expression of microRNA-625-3p is associated with poor response to first-line oxaliplatin based treatment of metastatic colorectal cancer. Mol. Oncol..

[76] Pardini B., Rosa F., Naccarati A., Vymetalkova V., Ye Y., Wu X., di Gaetano C., Buchler T., Novotny J., Matullo G. (2015). Polymorphisms in microRNA genes as predictors of clinical outcomes in colorectal cancer patients. Carcinogenesis.

[77] Boni V., Zarate R., Villa J.C., Bandrés E., Gomez M.A., Maiello E., Garcia-Foncillas J., Aranda E. (2011). Role of primary miRNA polymorphic variants in metastatic colon cancer patients treated with 5-fluorouracil and irinotecan. Pharm. J..

[78] Zhang Y., Geng L., Talmon G., Wang J. (2015). MicroRNA-520g confers drug resistance by regulating p21 expression in colorectal cancer. J. Biol. Chem..

[79] Pichler M., Winter E., Ress A.L., Bauernhofer T., Gerger A., Kiesslich T., Lax S., Samonigg H., Hoefler G. (2014). miR-181a is associated with poor clinical outcome in patients with colorectal cancer treated with EGFR inhibitor. J. Clin. Pathol..

[80] Cappuzzo F., Sacconi A., Landi L., Ludovini V., Biagioni F., D’Incecco A., Capodanno A., Salvini J., Corgna E., Cupini S. (2014). MicroRNA signature in metastatic colorectal cancer patients treated with anti-EGFR monoclonal antibodies. Clin. Colorectal. Cancer.

[81] Haggstrom D., Cheung W., Nekhlyudov L. (2015). Approach to the Long-Term Survivor of Colorectal Cancer.

[82] Yang X.D., Xu X.H., Zhang S.Y., Wu Y., Xing C.G., Ru G., Xu H.T., Cao J.P. (2015). Role of miR-100 in the radioresistance of colorectal cancer cells. Am. J. Cancer Res..

[83] Borralho P.M., Kren B.T., Castro R.E., da Silva I.B., Steer C.J., Rodrigues C.M. (2009). MicroRNA-143 reduces viability and increases sensitivity to 5-fluorouracil in HCT116 human colorectal cancer cells. FEBS J..

[84] Lee C.G., McCarthy S., Gruidl M., Timme C., Yeatman T.J. (2014). MicroRNA-147 induces a mesenchymal-to-epithelial transition (MET) and reverses EGFR inhibitor resistance. PLoS ONE.

[85] Ruzzo A., Graziano F., Vincenzi B., Canestrari E., Perrone G., Galluccio N., Catalano V., Loupakis F., Rabitti C., Santini D. (2012). High let-7a microRNA levels in KRAS-mutated colorectal carcinomas may rescue anti-EGFR therapy effects in patients with chemotherapy-refractory metastatic disease. Oncologist.

[86] Song B., Wang Y., Kudo K., Gavin E.J., Xi Y., Ju J. (2008). miR-192 Regulates dihydrofolate reductase and cellular proliferation through the p53-microRNA circuit. Clin. Cancer Res..

[87] Clark J., Grothey A., Goldberg R. (2015). Systemic Chemotherapy for Nonoperable Metastatic Colorectal Cancer: Treatment Recommendations.

[88] Bader A., Lammers P. The Therapeutic Potential of microRNAs. Innov. Pharm. Technol.

[89] Ling H., Fabbri M., Calin G.A. (2013). MicroRNAs and other non-coding RNAs as targets for anticancer drug development. Nat. Rev. Drug Discov..

